# Urban Green Space and the Pursuit of Health Equity in Parts of the United States

**DOI:** 10.3390/ijerph14111432

**Published:** 2017-11-22

**Authors:** Viniece Jennings, April Karen Baptiste, Na’Taki Osborne Jelks, Renée Skeete

**Affiliations:** 1 Southern Research Station, USDA Forest Service, 320 Green Street, Athens, GA 30602, USA; 2 Environmental Studies Program, Colgate University, 13 Oak Drive, Hamilton, NY 13346, USA; abaptiste@colgate.edu; 3 Department of Public Health, Agnes Scott College, 141 E. College Avenue, Decatur, GA 30030, USA; nosbornejelks@agnesscott.edu; 4 Department of Sociology, Georgia State University, 33 Gilmer Street SE, Atlanta, GA 30303, USA; renee.skeete@gmail.com

**Keywords:** nature, health equity, green space

## Abstract

Research has demonstrated that inequitable access to green space can relate to health disparities or inequalities. This commentary aims to shift the dialogue to initiatives that have integrated green spaces in projects that may promote health equity in the United States. Specifically, we connect this topic to factors such as community revitalization, affordable housing, neighborhood walkability, food security, job creation, and youth engagement. We provide a synopsis of locations and initiatives in different phases of development along with characteristics to support effectiveness and strategies to overcome challenges. The projects cover locations such as Atlanta (GA), Los Angeles (CA), the District of Columbia (Washington D.C.), South Bronx (NY), and Utica (NY). Such insight can develop our understanding of green space projects that support health equity and inform the dialogue on this topic in ways that advance research and advocacy.

## 1. Introduction

Health disparities, which pertain to major gaps in health related to factors such as race/ethnicity, socio-economic status, or geographic location, were formally recognized in the United States in 1985 [1], with chronic disease and injury-related mortality later emerging as key health concerns [2]. As entities documented the need to reduce health disparities, many acknowledge that these disparities relate to multiple factors in the social and environmental context [3]. For example, Braveman et al. [4] analyzed socioeconomic differences for 11 health indicators across the United States and found that many ailments were more pronounced in low-income and least-educated groups. Similar health disparities have been documented across the globe and in a number of industrialized countries [5] across North America [4,6] and Europe [7]. However, health disparities are not always consistent across sociodemographic groups and can be influenced by a range of factors such as sex, gender identity, geographic location, type of health outcome, and the causal pathways of manifestation [8]. Thus, there is room to better understand the underlying pathways that may mediate the relationship between socioeconomic disadvantage and public health [7]. Theories on social capital (e.g., extent and influence of social relationships), inequality (e.g., poorer health related to economic disparities and social injustice), and the political economy (e.g., diminished health from political and social exclusion from resources) have contributed to the public health scholarship [9].

**Figure 1:**
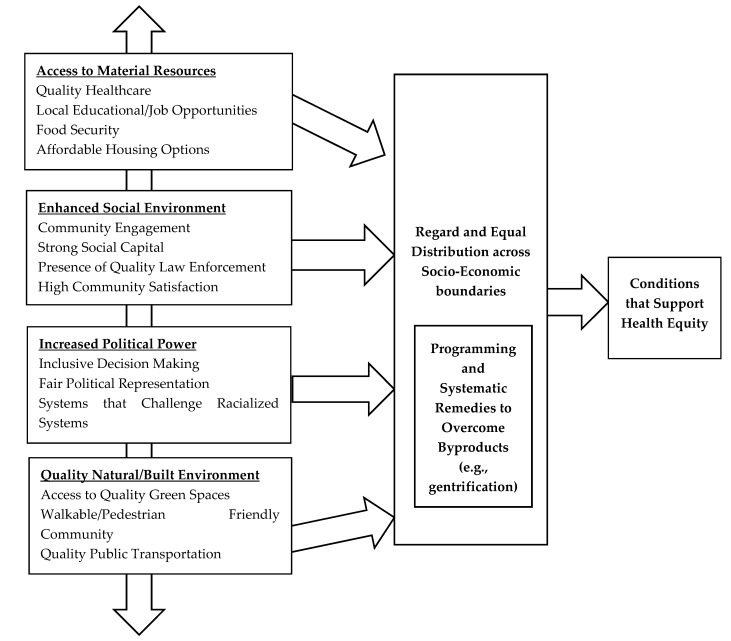
Conceptual framework that connects urban green spaces with factors that support health equity (Adapted from [12,13,14]).

Some scholars describe health equity as ‘social justice in health’ [8] and maintain that developing strategies to achieve health equity as a critical issue in the United States [10]. According to the U.S. Department of Health and Human Services, health equity is described as the “attainment of the highest level of health for all people. Achieving health equity requires valuing everyone equally with focused and ongoing societal efforts to address avoidable inequalities, historical and contemporary injustices, and the elimination of health and health care disparities” [11]. The traditional approach to reducing health disparities is to improve access to the medical care system. However, other structural forms of inequality relating to, for example, race, class, and political power, also influence disparities in health outcomes. Therefore, there are also a variety of mechanisms to increase health equity. Multiple articles [12] have used a model developed by Starfield and Birn [13] to assess whether and how interventions have achieved health equity through three primary mechanisms including: (1) improving access to material resources, (2) reducing chronic stress or enhanced physical and social environment, and (3) increasing political power. With this in mind, we developed the following conceptual model to illustrate the links between some factors involved in the relationship between green space and health equity (Figure 1).

Improved access to material resources can support individuals’, families’, and communities’ ability to afford basic necessities such as housing, access to nutritious food, job opportunities, and health care. Chronic stress is often experienced disproportionately in low-resource communities and linked to numerous poor health outcomes. Enhancing the physical and social environment is a key pathway that urban planning can improve health equity [14]. We also included community engagement, strong social capital, the presence of quality law enforcement, and high community satisfaction as some factors within quality social environments. These variables are important since a quality social environment relates to favorable health and the access to or use of resources such as preventive healthcare services [15]. Increased political power can assist communities in building and leveraging capacity to accomplish community goals. Quality features within the natural or built environment such as access to quality green spaces, public transportation, a walkable or pedestrian community, and a distributive layout of environmental burdens.

Characteristics of place, such as the presence of green space, can play a key factor in the interaction between poverty and health [16], environmental justice [17], and health equity [18]. It can be challenging to define the quality of green spaces since it interfaces the needs of humans and tree cover, relates to the specific ecosystem service and/or health outcome of interest, and is influenced by the aesthetic expectations of green spaces, which is often subjective to an individual and may not necessarily reflect general preferences. However, a broad description of quality green spaces can include those that are well-maintained, properly configured to support tree health, and designed in a way that appeals to urban dwellers. Some cities use the coverage of tree canopies as an indicator of environmental injustice [19]. Likewise, social determinants of health (e.g., neighborhood and built environment) play a foundational role in health equity as they can support changes in the environmental context and other factors involved in health promotion [14]. To elaborate, a study in Philadelphia compared the heart rates of study participants with a view of non-greened vacant lots versus views of greened vacant lots, and found that green lots may significantly reduce stress in neighborhoods [20]. Also, the benefits from green space can relate to other social determinants of health (e.g., education, neighborhood and built environment, economic stability, etc.) in numerous ways [21]. For these reasons, we will focus on the role of urban green spaces in this article.

Initiatives on urban green space can support the goals of enhancing human well-being [22], reducing health disparities [23], improving community health and safety [16], increasing residential empowerment [24], and encouraging sustainable communities [25]. For instance, Collins et al. [6] surveyed politicians and senior members of local government in metro-Vancouver (Canada) where respondents perceived that investing in parks and recreational facilities was a key way to address health inequities. While the perception and benefits from green space can vary, these observations are influenced by factors such as the type, local environment, and maintenance of vegetation [16,26]. Understanding the role of urban green spaces on equity and social determinants of health can also inform our efforts to advance sustainable development [21] and inform policies related to health equity from a socioeconomic perspective [4].

The relational approach to achieve health equity recognizes the interaction between place and social processes, which collectively influence human health [27]. However, few studies have linked findings from the research community with the interventions from municipal governments [6] or others involved in the movement to achieve health equity. As a result, it can also be beneficial to discuss the potential role that interventions play in minimizing consequences to public health [7].

In this commentary, we extend the dialogue on social and health equity in the United States through projects related to the social benefits of urban green space. Specifically, we draw on examples to illustrate how green spaces can contribute to a quality urban environment in ways that support health equity. This discussion elevates the role of urban green spaces and programs in their relationship to social determinants of health and pursuit of health equity. The initiatives included in this commentary were selected from an interdisciplinary approach based on the author’s perspectives and research. We relate this topic to potential benefits from urban green spaces and issues such as local job creation, affordable housing, walkable communities, and combating inequitable access to healthy foods. We conclude with recommendations to guide future research and initiatives that aim to promote health equity through urban green spaces. To our knowledge, this article represents one of the first documents that discuss the role of green space and the collective vision of promoting health equity.

## 2. Urban Green Space Projects and Initiatives to Support Health Equity in the United States

### 2.1. Green Space and Affordable Housing: The East Lake Community (Atlanta, GA)

The quality of housing and built environment is an important health indicator as it relates to equity in living conditions [28]. Improving housing conditions is an avenue to enhance the physical environment where people reside. However, income often drives the home selection process, leaving low-income households with few choices outside of neighborhoods with hazardous characteristics [14]. Housing can contribute to health in various ways, including through its role in building social networks [29], levels of physical activity [29,30], and health-related behaviors such as smoking [31]. The provision of high-quality affordable housing is a key way for municipalities to address health disparities [6]. Some redevelopment efforts have successfully employed green space redevelopment as a strategy to promote healthier affordable housing in healthier neighborhoods [27]. In this section, we highlight the redevelopment of East Lake Meadows in the City of Atlanta.

East Lake Meadows, with approximately 650 units of public housing, was constructed in 1970, at the tail end of the public housing construction boom [32]. Thomas Cousins, a developer and philanthropist, spearheaded the plan to revitalize the East Lake community after learning the connection between neighborhood conditions and crime [33]. He bought the East Lake Country Club, a 177-acre property with a renown golf course and three lakes, when it became available for sale in 1993 [34]. That green space purchase was the foundation for the transformation process. The absence of an overarching organization to implement the project led Cousins to establish the East Lake Foundation to fuel the revitalization effort through a comprehensive and multipronged approach to community development [34]. Profits from the East Lake golf course, over $20 million to date, were directed to fund the East Lake Foundation [35]. Seven institutions constitute the East Lake Campus: the apartment complex, charter school, early childhood education center, golf club, golf course, YMCA, and the East Lake Foundation [36].

In partnership with the Atlanta Housing Authority, which retains ownership of the ground beneath the redevelopment, the East Lake Foundation utilized a combination of federal funds and East Lake Foundation funds to demolish East Lake Meadows and build the Villages of East Lake [37]. The new complex features a 50/50 mix of 542 public-housing-eligible and market-rate units, [33,38] which is a much higher ratio of subsidized units than the typical 20% in most mixed-income redevelopments [39]. Prospective residents interested in the mixed-income apartment community must undergo a criminal and credit background check during the application process, which can have its share of advantages and disadvantages. The Villages of East Lake development boasts a number of programs and services to enhance the residential experience and facilitate economic self-sufficiency for the residents who utilize housing subsidies, including: early childhood education, a before- and after-school program, a golf mentorship program for youth, tutoring and career development assistance, and other resident socials and classes [33,36].

Perhaps most notably, East Lake is the home of the Charles R. Drew Charter public school, which is known for its strong curriculum and pedagogy. The school has played a key role in the redevelopment’s success, as it has been a major draw for new neighborhood residents [40]. The Charles Drew charter school is one of the top-performing schools in the city with a graduation rate of 78%, which is considerably higher than its 30% graduation rate before redevelopment [41]. Golf programs at the Charlie Yates Golf Course are integrated into the physical activity curriculum at Drew, allowing participating students to make full use of the community green space [42].

Although the East Lake redevelopment was largely an economic and social success, it was not without its challenges and limitations. While the neighborhood remained accessible to the proportion of original residents who were able to return after redevelopment, the same cannot be said with certainty for residents who did not meet the return criteria and for homeowners and renters in the neighborhood surrounding East Lake Meadows. As home values increased at a rate four times greater than in the average increase for Atlanta [42], fixed- and low-income homeowners and renters suffered net losses as a result of rising property values [38]. Overall, the community has done remarkably well in terms of low-income residents having access to the gains the redevelopment has produced [43].

The East Lake Foundation identified five steps to transform neighborhoods: (1) carefully select the neighborhood (look for the right scale where reasonable impact can take place), (2) identify a champion (include a visionary on the team that values and operates with a collaborative approach), (3) define the initiative in a way that acknowledges the importance of both housing and education, (4) create a funding plan (e.g., low income housing tax credits and private investment) that considers insight from for-profit and non-profit entities, and (5) make the commitment for the long term [41,44]. This project illustrates how a green space (i.e., golf course) can not only provide a recreational amenity, but also serve as a powerful catalyst to transform the surrounding community [41]. By drawing on East Lake’s heritage as a recreation-oriented neighborhood, this green space redevelopment project has established the East Lake neighborhood of Atlanta as a desirable, amenity-rich community with improved resident access to economic, educational, and recreational resources that can create a foundation for physical and mental health. By providing and maintaining access to health-promoting resources for some low-income residents of East Lake, the redevelopment project demonstrated the possibility for promoting health equity through green space redevelopment. This intervention goes beyond the typical method of improving access to individual services to addressing the ecosystem of the neighborhood as a whole, as Jutte et al. recommend [45]. While direct measures of health outcomes for East Lake residents are, unfortunately, unavailable, the East Lake Foundation engaged several of the social determinants of health in this greenspace-centered, multifaceted intervention. The redevelopment with the East Lake golf course as a catalyst yielded improvements in the quality of the housing stock, education, recreation facilities, food access, and employment opportunities. However, the redevelopment also spurred rapid displacement [38,42], which can have negative impacts on the displaced and on lower-income residents who remain in the neighborhood as it transitions [46].

### 2.2. Youth Engagement and Urban Greening: Los Angeles, CA

Favorable experiences in urban nature, especially during formative years, can develop values that relate to environmental stewardship and advocacy. Metcalf et al. [47] recently described how youth engagement in urban greening efforts represents a social movement that plays a critical role in achieving health equity. Specifically, programs that engage youth in urban greening efforts provide a mechanism for the following benefits: greater exposure to green space, increased opportunities for social interaction and connectedness, mentorship, educational opportunities, a greater sense of self and psychological resilience, and a favorable perception for control in life [47]. Similarly, an empirical study documented how outdoor youth programs are an avenue to significantly enhance social development, sense of community, and self-efficacy [48]. Given modern public health concerns among adolescents related to bullying, violence, depression, and obesity [49], along with the potential for urban green space to buffer health disparities, merging the dialogue to include youth engagement is important. This is also critical since aspects of individual empowerment and ‘control over destiny’ can influence health in general yet be particularly critical in underserved communities and across other socioeconomic boundaries [50]. Thus, the benefits of youth engagement in urban greening can relate to an enhanced social environment and access to resources that support health equity. In this section, we will discuss youth engagement and urban greening activities in Los Angeles, California.

Los Angeles has had its share of health concerns, particularly in minority and low-income communities [51]. Despite the lack of funding for quality after-school programming in Los Angeles County, green spaces such as parks provide a great resource to support underserved communities and promote public health [52]. According to its website, Los Angeles is home to the nation’s oldest and largest urban conservation Corps and it aims to provide “work experience with an emphasis on conservation and service projects that benefit the community” [53]. For example, Pincetl [54] documented the role of various partners involved in the Los Angeles Million Tree Initiative and noted that the LA Conservation Corps served a primary role in the city’s tree planting programs. Along with the Conservation Corps, other entities are involved with youth engagement and urban nature in Los Angeles. The Natural Leaders Initiative is a program under the Children and Nature Network (C&NN) and it aims to develop a diverse cadre of young leaders (between the ages of 18 and 29) who work to promote equitable access to green spaces in their communities [55]. As of 2017, C&NN has recruited nearly 200 young leaders from across the United States [55] and has about 24 Natural Leaders in and around the Los Angeles area who have supported a range of positive impacts throughout the city (personal communication CJ Goulding, Lead Organizer, Natural Leaders Network, February 2017). For example, Little Green Fingers is a program within the L.A. Conservation Corps which supports community gardens throughout low-income neighborhoods [56]. An alumna of the Natural Leaders Network serves as a program manager of this program and under her leadership the number of gardens has nearly doubled and continues to serve the diverse residents of East Los Angeles (personal communication CJ Goulding, Lead Organizer, Natural Leaders Network, February 2017). Juan Martinez, a native of Los Angeles, is C&NN’s Director of Leadership Development, where he oversees programming to develop millennials in community organizing and other aspects of cultural capacity to mobilize the movement of engaging under-represented groups in the outdoors [57]. The Natural Leaders Initiative also partners with other entities to organize Fresh Track Leadership Expeditions, which is a program that involves outdoor exploration as an avenue to enhance cultural competency, civic engagement, hometown stewardship, and workforce development [58]. In a report to evaluate the Fresh Tracks program, participants were involved in a pre- and post-trip survey which showed an increase in all outcomes related to this initiative [58]. Consequently, programs that engage youth in urban greening stewardship, especially in underserved communities, provide benefits that should continue to be developed in research and advocacy [47]. Along with these efforts in Los Angeles, other initiatives to engage youth and young adults in the outdoors include organizations such as Groundworks USA, Outdoor Afro, and Kids in the Woods program by the U.S. Forest Service. In addition, the Healthy Parks Healthy People Initiative, which is led by the National Park Service, as well as other federal partnerships are heavily involved in these efforts [59]. Along with youth engagement, others have explored equity in green space access in the Los Angeles area [60]. Thus, efforts to develop local leadership for urban greening goals can promote health [47], develop advocates for environmental stewardship, and increase ones exposure to opportunities that can enhance their outlook on life.

### 2.3. Green Space and Local Job Creation: South Bronx and Atlanta

Many studies affirm the positive role of urban green spaces to increase property values [61], revitalize communities [62], and enhance public health safe-guards, particularly in vulnerable populations [63]. Others note that greening initiatives can also contribute to green collar workforce development [16,64] through training and employment opportunities, thereby providing a pathway to health equity by improving access to material resources [12,13]. While some studies document how parks and other public lands provide youth training and work opportunities [27,65], few have discussed their economic impact or have explicitly discussed their role in promoting health equity. This section highlights urban green space projects as an avenue for job creation in South Bronx, New York and Atlanta, Georgia.

#### 2.3.1. Sustainable South Bronx (SSBX)

Over time, environmental injustice and economic deprivation have been documented in the South Bronx [66], especially in relation to concentrated industrial sites, health disparities, public safety issues, and poverty [67]. With the support of a seed grant and mayoral appropriation, Sustainable South Bronx (SSBX) received about three million dollars to support the Hunts Point Riverside Park—the area’s first waterfront access point in 60 years [66,67]. Collectively, this four-mile greenway improved access to parks (i.e., Hunts Point Riverside Park and Barreto Point Park) and promoted physical activity through bicycle- and pedestrian-friendly infrastructure [67]. Project partners also worked to improve traffic signaling for pedestrians, increased police presence at the parks, and collaborated with a local congressman’s office to facilitate park maintenance [67].

In 2003, SSBX founded the Bronx Environmental Stewardship (BEST) Academy which trains low-income residents in NYC for environmental jobs by teaching multiple skills used to restore urban green spaces [68]. Graduates from the BEST Academy are also eligible to work in SSBX’s Smart Roofs program that supports the city’s Go Green initiative by constructing green roofs [68]. Both the greenway project and SSBX’s programs to create green roofs and restore urban green spaces have increased access to open space and provided an avenue for economic development [66]. The program also involved a youth job training program through which participants planted 400 trees and helped with maintenance and environmental education [67]. BEST serves as a model in community revitalization that engages residents in confronting health disparities and environmental injustice in ways that are sustainable and fiscally sound [69].

Since its inception, SSBX has trained more than 500 low-income New York residents to work in the growing green collar sector through these combined initiatives [68]. Since 2008 alone, 150 graduates have completed the BEST training program. Eighty-five percent of the graduates obtained employment after graduation, with more than 90% employed in green collar jobs and nearly 10% pursuing a college track [70]. Although a recent study observed fewer vacant or abandoned buildings and litter, there is room for more work to be done on fully achieving environmental justice in the South Bronx [71]. Today, other parts of New York City have outlined efforts to promote neighborhood health through urban parks [72], making Sustainable South Bronx an inspiration for others. Thus, urban greening projects can support local workforce training and employment opportunities that broaden social networks as well as facilitate personal development and empowerment.

#### 2.3.2. Proctor Creek (Atlanta, GA)

The Proctor Creek Watershed is an environmentally degraded urban watershed plagued by multiple pollution stressors and storm water management challenges [73]. The English Avenue neighborhood is one of 38 neighborhoods in Northwest Atlanta’s Proctor Creek Watershed, and it has suffered from high crime, unemployment, and divestment of public resources in infrastructure [74]. English Avenue is also the most under-parked area within the city of Atlanta [74]. In 2013, the Urban Waters Federal Partnership designated the watershed as a priority site for concerns related to sewer overflow discharges, other pollution, and erosion [75]. At the invitation of community leaders, a local parks advocacy organization, Park Pride, led a community-engaged visioning process for a series of parks and green infrastructure (GI) elements in the upper reaches of the Proctor Creek Watershed.

Construction of the 1.5-acre Lindsay Street Park involved a year-and-a-half-long paid training and work experience program for young adults from the English Avenue and adjacent Vine City neighborhoods [73]. These young adults (ages 18–24) were trainees in the Atlanta Youth Corps (AYC), a program affiliated with the non-profit, Greening Youth Foundation (GYF), a group whose mission is to “work with diverse, underserved, and underrepresented youth and young adults in an effort to develop and nurture enthusiastic and responsible environmental stewards” [76]. Trainees gained skills in construction, gardening, landscaping, and other knowledge for installing GI features such as rain gardens, bioswales, and bioretention areas [73]. Along with the AYC crew, more than 20 additional residents from Build-Up, a social-venture program established by Georgia STAND-UP, led the “green” demolition of a dilapidated property to prep the site for the park [73].

Following the example of Lindsay Street Park, project partners, including Park Pride, The Conservation Fund, West Atlanta Watershed Alliance, GYF, and others, are developing a new green infrastructure park in the Proctor Creek Watershed, named Boone Park West. This new park project was one of four 2016 recipients of the American Planning Association (APA) and the National Recreation and Park Association’s Great Urban Parks Campaign grants. This joint initiative “… aims to improve environmental and social outcomes in underserved communities through green infrastructure projects in local parks. Additionally, the Campaign will result in the development of training resources for park, planning and green infrastructure professionals to improve equity through green infrastructure” [77]. The projects in Proctor Creek contribute to the city’s commitment to sustainability, which is reflected in its Green Infrastructure Strategic Plan and a post-development storm water management ordinance [78]. Boone Park West and other new green infrastructure projects in the Proctor Creek Watershed, including the Boone Boulevard Green Street [79] are slated to include workforce development opportunities for local residents.

Other green space projects in the U.S. are also involved in job creation opportunities and are responding to growing demands for certified green infrastructure professionals. In 2017 the National Green Infrastructure Certification Program, a collaboration of DC Water and the Water Environment Foundation (WEF), graduated and certified over 100 participants from key pilot cities in the Mid-Atlantic and Mid-West regions of the United States [80]. The program is slated to be launched nationally in 2018 [80]. Although other green space programs also represent emerging models for job placement in the U.S. [81], few have been identified in academic literature [81]. Nonetheless, these initiatives offer promising models to enhance employment options in underserved populations as well as improvement of environmental quality and health in environmentally degraded communities [12].

The increase of accessible urban green space in environmentally degraded and economically disadvantaged communities is important for the promotion of physical and mental health [82]. The opportunity for community residents to gain job skills and employment through greening projects is also important in advancing health equity. Many of these initiatives in places like Atlanta, Georgia are in early stages; making their true impact difficult to measure. Those such as SSBX’s BEST Academy have more longevity, however empirical research about them is also limited. As additional knowledge about the long-term effectiveness of these programs can be obtained over time, the extent of their impact on reducing the health equity gap can be better evaluated.

### 2.4. Walkable Neighborhoods and Green Space: Washington D.C.

As the U.S Surgeon General recently made a national call of action to promote walking communities [83], this topic is discussed in the pursuit of health equity. The aesthetic value and benefits from urban green spaces can enhance the physical and social environment. For instance, green spaces can provide opportunities to increase physical activity and enhance aspects of place which relate to well-being through social cohesion and community satisfaction [21]. Some regard the District of Columbia (D.C.) as a national model for walkable urban spaces [84]. According to Leinberger [84], D.C. is the only metropolitan area in the United States that includes six types of walkable urban place that allow urban form to meet function: (1) downtown, (2) downtown adjacent, (3) urban commercial, (4) suburban town center, (5) strip commercial redevelopment, and (6) greenfield. Parks and other types of green space are also mixed into residential/commercial development in different parts of the city [84]. In 2013, Washington D.C. contained roughly 22% percent parkland, ranking it among the top high-density cities for parkland in the U.S. [85]. In its efforts to improve pedestrian mobility, D.C.’s Iona Senior Services is considered a community success story that took a number of steps to improve its pedestrian experience [86]. Specifically, they conducted a walkabout in which they collected data, focused on accessibility, raised funds, and promoted changes to policy or engineering decisions [86]. In 2012, the city also launched the Play DC initiative which is a multi-year effort to renovate play spaces across the city [87]. According to 2010 data, about 86% of the Black and Hispanic population in the District of Columbia live within a half mile of a park [88].

While safe walking spaces are also important in health promotion [25], a survey of parents in the Washington D.C. metro area reiterated the importance of walkability, safety, areas for active play, and neighborhood aesthetics—particularly for active children [89]. Previous research in D.C. neighborhoods observed a greater fear of crime with age and female gender, however, the length of time in a same neighborhood was linked with less concerns about crime [90]. Even though parents of active children in the D.C. area agreed on the importance of the built environment on physical activity, they also reported higher crime rates and being a victim of neighborhood crimes yet crime was not a hindrance to active play among children [89]. Roberts et al. [89] suggested that these parents may utilize other safety measures to ensure that crime does not barricade their child’s physical activity levels. On the other hand, a report which includes a focus group of Hispanic pedestrians in D.C. noted the role of cultural differences and language barrier that results in pedestrian and bicycle safety concerns for this community [91]. 

Although racial and ethnic disparities in levels of active play exist in Washington D.C. [89], a number of initiatives are making strides to tackle this issue. For instance, during 2016, the Let’s Move Outside Initiative included over 90 events and 4000 volunteers throughout the Washington D.C. area along with 380 events in Baltimore and over 8000 volunteers (Personal Communication with Tracy Muckey, 13 January 2017, YMCA—Washington D.C.). Under the direction of Dr. Robert Zarr, the D.C. chapter for the American Academy of Pediatrics has a Park Rx program where physicians prescribe time outdoors as a strategy to promote physical activity and health promotion [92]. Dr. Zarr, who has hundreds of low-income and immigrant patients, has also developed an online portal of 350 green spaces throughout D.C. to guide physicians in promoting accessible and safe places to exercise [93]. National organizations such as Girl Trek also have a strong presence in the D.C.–Maryland area [94] and serve a model for integrating social media with health interventions to promote walking to help reduce health disparities in minority communities [95]. Through its website (www.girltrek.org), Girl Trek uses an online platform to organize and announce year-round group walking events that are particularly geared to African American women [96]. Also, FitDC, an initiative with the city’s Department of Health in addition to its Department of Parks and Recreation, includes a team of diverse coaches who represent different wards throughout the city and lead monthly ‘Ward Walks’ to encourage residents [97]. Data for Washington D.C. between 2011 and 2013 note that the percentage of Blacks and Hispanic persons who participated in 150 min or more of physical activity per week increased [98]. Notably, the percentage level of physical activity among Hispanics has increased by roughly 11% [98].

According to their website, groups such as All Walks DC advocate for a number of pedestrian issues around safety, adequate design, and engaging the public in transportation concerns (allwalksdc.org). On the other hand, the type of walkability (e.g., recreational or utilitarian purposes) can also affect the role of urban green spaces such as parks [99]. A study in D.C. considered visiting parks as a social-entertainment mode of walking which is influenced by the built environment, however, utilitarian forms of walkability maybe more influenced by neighborhood location and access to public transportation instead of access to parks [99]. Even though this section did not focus on one specific organization in D.C., this describes how creative strategies in public engagement and research inquiry have not only encouraged walkability but also empirically explored public perspectives to overcome barriers. As green spaces generally encourage greater levels of health promoting behaviors such as walking [100], their aesthetic appeal can enhance the environmental and social context of cities in ways that support health equity.

### 2.5. Urban Gardens and Access to Healthy Food Options in Utica, New York

In recent years, research on equitable access to healthy foods has received growing attention in the United States [101]. Some describe ‘food deserts’ as areas characterized by high concentrations of low-income residents, fast food franchises, and limited sources of fresh foods [102]. Inequitable access to quality sources of food can lead to health inequalities related to nutrition in both high- and low-income cities [103]. Despite the mixed observations between proximity to healthy foods, eating habits, and health, studies across North America regularly demonstrate that communities dominated by low-income or minority persons tend to have less access to healthy foods options [104]. A city’s capacity to provide access to healthy foods relate to the vision of achieving health equity as well as environmental justice [105]. Some initiatives are engaged in efforts for food provision or deeper empowerment goals within marginalized communities, focusing on food justice and food sovereignty [106]. Many grassroots organizations promote urban gardens in these endeavors. Community gardening can promote neighborhood attachment which enhances one’s bond to the physical and social environment [107]. Based on content analysis from written materials, internet sources, and an interview with its president, this section will discuss For the Good Inc., an organization that seeks to combat food concerns as well as deeper community issues in Utica, NY.

Utica has experienced its share of impoverishment with the average income being 14% lower than the state average, 17% of its population relying on food assistance programs, and over 30% in poverty [108]. For the Good Inc. (Utica, NY, USA) (FTG) a registered non-governmental organization that seeks to provide low-income residents with programs to overcome poverty [109]. Since its inception, the organization has been engaged in numerous community interventions including crime reduction to establishing urban gardens [109]. The first garden was built in 2008 with the help of the city of Utica, Wal-Mart, Cornell University, and a local benefactor [109], the organization was encouraged to expand the gardens to include two additional locations. These gardens provide fresh vegetables, a space to promote physical activity as well as community building, and community beautification by converting abandoned lots into green spaces, which pertains to a quality natural or built environment.

Vegetables from the garden are given to volunteers as well as others in need. The organization has also built partnerships with local agencies who supply labor for the garden. Ms. Harris-Lockwood was particularly excited about a win-win partnership with the Refugee Center of Utica, which is affiliated with the county’s youth program, which has taken place over the past two years [110]. Ms. Harris-Lockwood also expressed that a partnership with the local city service providers, such as the fire department, was also crucial to the urban garden program. Also, involving a local resident to manage the urban garden is an avenue for the program to develop trust with community members and affords a way to recruiting both volunteers and permanent participants who will support the garden ascribing to the another factor of an enhanced social environment. For example, strong relationships with the local government have allowed For the Good to access land resources as well as abandoned or empty lots to continue their work [109]. In particularly depressed urban areas, like Utica, this allows for abandoned spaces to be put to one form of productive use. Scholars have shown that the use of urban and community gardens tend to have positive effects on neighborhoods.

Local challenges to For the Good’s garden initiative include obtaining funding for staff and equipment, improving how residents’ embrace local produce, and also locating an appropriate manager for the garden [110]. FTG is regularly seeking out new grants to support the overall operation of the organization, which includes the garden project. Further, attempts to change food perceptions and preferences must be done in a measured manner. Particularly in the city of Utica where there is a growing refugee population, cultural appropriateness of food, as encouraged by food justice advocates [111], is key to influencing perceptions. This is a challenge that FTG and other food justice organizations will need to take on in the near future research.

The organization has attempted to address its challenges in several ways. For instance, the following factors have supported the progress made with the For the Good’s garden initiative thus far: community support, seed drives from local businesses, multiple organizational models to ensure a sustained labor force, hiring a local resident to manage the garden, and strong relationships with different stakeholders [110]. “I don’t know about every year, but the last two or three years we’ve gotten a wonderful donation from Wal-Mart, and whatever seeds they don’t sell they give to us. We end up giving away tons of seeds [110].”

Many urban areas that are faced with an exodus of economic activity are faced with blight and other social problems. Green spaces then act as a way to give community members a way to enhance their physical spaces. Levkoe [112] highlighted in his work on The Stop in Toronto, the ways in which community gardens can bring community members together around food encouraging healthier eating lifestyles but additionally provides a space for community members to engage in physical activity. Organizations like For the Good struggle with many issues, such as funding, staffing capacity, and getting community involvement at times. Yet, as Ms. Harris-Lockwood has showcased, the work is extremely important in places where access to fresh fruit and vegetables may be limited. The initiative in Utica attempts to show that urban areas that have an exodus of economic activity tend to suffer from environmental injustices as access of food in the form of full-scale grocery stores are often not available. Having urban gardens like those promoted by FTG begins to create spaces where urban communities can get access to fresh fruits and vegetables even if on a limited scale, hence beginning to scratch the surface of health disparities as it relates to food access.

## 3. Closing Remarks and Looking Forward

The pursuit of health equity involves a process over time. Hence, we do not claim that these projects and/or locations are the sole examples to consider or that they do not have potential areas of improvement. Rather, they illustrate how green spaces can relate to health equity. As green spaces play a role in place-based social or health initiatives, we acknowledge that other factors (e.g., transportation, access to quality health care) influence health outcomes. For example, other design elements such as adequate sidewalks, public transportation, lighting, and cross walks can enhance walkability and levels of physical activity. Moreover, even if residents consider a crime-stricken area to be relatively safe during the day [113], others maintain that unresolved crime issues can limit efforts to improve neighborhood walkability [114]. Behavioral interventions (e.g., to increase physical activity) may not be effective for people who are faced with other concerns (e.g., poverty, disability, racism) that greatly burden their well-being [6]. To demonstrate, a focus group study interviewed racial or ethnic minorities in the South Bronx and found strong concerns about second-rate health care, distrust, racism, and feeling undervalued by the local healthcare system [115]. On the other hand, when it comes to gardening projects, some note that the issue of soil contamination should also be considered in urban gardening efforts [116], which can be particularly important in blighted areas.

The aforementioned projects discuss how some components with our conceptual model can link outcomes of urban greening efforts to health equity. For example, the initiative in East Lake utilized a housing subsidy program to support the inclusion of low-income residents in its development process. In Utica, initiatives demonstrated that urban gardening projects can provide an avenue for marginalized communities to get access to fresh fruits and vegetables which are important for proper nutrition. However, other ways to systematically overcome gentrification remains a challenge and presents a prospective area of research. In addition, we provide the following recommendations to guide research and practice:

- Perform a case study analysis to assess the best practices, management techniques, strategies to quantify health impacts, and recommendations from urban greening efforts in diverse communities;
- Analyze city policies and programs that may help to combat gentrification;
- Develop more longitudinal studies on urban gardens to identify the type of users, food preferences and perceptions, and changes in dietary behavior that support a nutritious lifestyle;
- Execute qualitative research methods to understand how cities are overcoming the challenges related to gentrification and identify entities that will help address this concern;
- Quantify how such initiatives allow youth to enhance their educational or job opportunities in ways that support empowered decision-making;
- Encourage the development and use of new datasets to monitor the changes in health outcomes within diverse population at multiple scales (e.g., city, neighborhood, block group, etc.);
- Improve how we quantify the role of urban greening efforts to determinants of health such as local employment opportunities, residential retention, and improved social interactions in underserved communities;
- Evaluate green workforce development programs to understand their effectiveness in improving access to material resources and full-time employment in diverse- or low-income communities.

## 4. Conclusions

Efforts that identify effective practices and provide critical perspectives to achieve health equity can inform research and programming. While some efforts use health impact assessments, integrating this insight with such efforts can inform the implementation of urban greening projects to effectively support health promotion. Hence, effective teams that are conducive to transdisciplinary and multi-sector partnerships are important in health equity initiatives. Along with the partnerships (e.g., local, federal, and private) shared in these projects, others note that the New Markets Tax Credit maybe another avenue to assist with park and recreation efforts in low-income communities [117]. Tsui [12] recommends that government and other entities join forces to create career pathways in growth industries to ensure that high-quality, career ladder jobs are available for graduates at the conclusion of their workforce training programs. Initiatives that recognize the importance of green space conservation and their equitable access in diverse communities can reinforce values from environmental ethics and social justice which are vital in health promotion [118] and preventive medicine [119]. Another resource that can complement green space projects include the Parks, Trails, and Health Workbook which was developed by the Centers for Disease Control and Prevention (CDC) and National Park Service. Scholars have also developed mapping applications to monitor environmental stewardship [120] and equity concerns [121]. Conversely, as some low-income residents expressed concerns about gentrification, it is critical that projects to expand urban green spaces are community-oriented, have a social cause, and provide support to maintain such initiatives in an equitable way [122]. Such efforts can support the pursuit of social justice, health equity, and sustainability in diverse urban communities.
